# Recombinant Analogs of Sea Anemone Kunitz-Type Peptides Influence P2X7 Receptor Activity in Neuro-2a Cells

**DOI:** 10.3390/md21030192

**Published:** 2023-03-20

**Authors:** Evgeny A. Pislyagin, Ekaterina S. Menchinskaya, Irina N. Gladkikh, Aleksandra N. Kvetkina, Oksana V. Sintsova, Darya V. Popkova, Sergei A. Kozlovskiy, Tatiana Y. Gorpenchenko, Galina N. Likhatskaya, Leonid A. Kaluzhskiy, Alexis S. Ivanov, Yaroslav A. Andreev, Sergey A. Kozlov, Pavel S. Dmitrenok, Dmitry L. Aminin, Elena V. Leychenko

**Affiliations:** 1 G.B. Elyakov Pacific Institute of Bioorganic Chemistry, Far-Eastern Branch of the Russian Academy of Science, 690022 Vladivostok, Russia; 2 Michael Sars Centre, University of Bergen, 5020 Bergen, Norway; 3 Federal Scientific Center of the East Asia Terrestrial Biodiversity, Far Eastern Branch of the Russian Academy of Sciences, 690022 Vladivostok, Russia; 4 V.N. Orekhovich Institute of Biomedical Chemistry, 10, Pogodinskaya St., 119121 Moscow, Russia; 5 Shemyakin-Ovchinnikov Institute of Bioorganic Chemistry, Russian Academy of Sciences, ul. Miklukho-Maklaya 16/10, 117997 Moscow, Russia; 6 Institute of Molecular Medicine, Sechenov First Moscow State Medical University, Trubetskaya Str. 8, Bld. 2, 119991 Moscow, Russia

**Keywords:** P2X7 receptor, Ca^2+^ influx, dye uptake, molecular docking, Kunitz-type peptides, neuroprotective activity, sea anemones

## Abstract

Purinergic P2X7 receptors (P2X7) have now been proven to play an important role and represent an important therapeutic target in many pathological conditions including neurodegeneration. Here, we investigated the impact of peptides on purinergic signaling in Neuro-2a cells through the P2X7 subtype in in vitro models. We have found that a number of recombinant peptides, analogs of sea anemone Kunitz-type peptides, are able to influence the action of high concentrations of ATP and thereby reduce the toxic effects of ATP. The influx of calcium, as well as the fluorescent dye YO-PRO-1, was significantly suppressed by the studied peptides. Immunofluorescence experiments confirmed that the peptides reduce the P2X7 expression level in neuronal Neuro-2a cells. Two selected active peptides, HCRG1 and HCGS1.10, were found to specifically interact with the extracellular domain of P2X7 and formed stable complexes with the receptor in surface plasmon resonance experiments. The molecular docking approach allowed us to establish the putative binding sites of the most active HCRG1 peptide on the extracellular domain of the P2X7 homotrimer and propose a mechanism for regulating its function. Thus, our work demonstrates the ability of the Kunitz-type peptides to prevent neuronal death by affecting signaling through the P2X7 receptor.

## 1. Introduction

Purinergic receptors presented in different cells of living organisms belong to a family of ligand-gated receptors that can be divided into ionotropic and metabotropic ones. Ionotropic purinergic P2X receptors are ATP-triggered homo/heterotrimeric ion channels that are required for various physiological processes, including immune system response, pain perception, and programmed cell death. The P2X7 receptor subtype has both structural and functional differences from other P2Xs. For example, its sensitivity to ATP is much lower, which gives it the role of a “noxious signal” detector of high concentrations of extracellular ATP, the release of which should be associated with cell death and tissue damage [1]. At the same time, the activation of P2X7 not only generates a non-selective cation influx but allows the passage of large molecules through the cellular membrane, such as fluorescent dyes such as YO-PRO-1, since the pore size reaches 8.5 Å in diameter. The use of YO-PRO-1 low molecular weight fluorescent compound is a proven method in studying the permeability of macropores formed by the P2X7 receptor [2].

The role of P2X7 in inflammation, cancer and the immune response was well-studied and recognized [3]. Some studies have previously confirmed that P2X7 is the main participant in the processing and release of pro-inflammatory IL-1β, and the receptor blocking leads to an improvement in the disease in various experimental models, such as inflammation from various infections, autoimmune diseases, models of tissue or organ damage, as well as neurological diseases. [4,5]. P2X7 also plays an important role in several diseases of the central nervous system development such as neurodegenerative disorders including Parkinson’s, Alzheimer’s, and traumatic brain injury [6,7].

Intracerebroventricular administration of the non-selective P2X receptor antagonist PPADS reduced infarct volume and the number of irreversibly damaged cells in the brains of spontaneously hypertensive rats [8]. Intrathecal administration of the P2X7 antagonist A438079 suppressed the development of mechanical hypersensitivity in the hind paw ipsilateral to the nerve injury. Activated P2X7 has also been reported to alter reactive oxygen species (ROS) in microglia/macrophages as was shown, for example, in primary rat microglial cells [9]. In cultured neurons of the dorsal horn of the spinal cord, ATP or Bz-ATP-induced ROS production was eliminated by both the ROS scavenger N-tert-butyl-α-phenylnitrone and the P2X7 antagonist A438079. Therefore, a comprehensive study of the P2X7 functions and search for its selective inhibitors will provide a better understanding of the physiological role of the receptor in a variety of conditions, as well as help in the development of new therapeutic agents to combat many socially significant diseases.

Marine organisms, especially venomous animals, represent inexhaustible but insufficiently studied sources of biologically active compounds for drug development [10]. One of them is sea anemones, whose venom represents a complex of biologically active proteins and peptides capable of influencing various molecular targets including serine proteases, ion channels and receptors as well as producing cytolytic effects [11]. Recent studies have shown that the major compounds of sea anemone venoms, along with neuro and cytotoxins, are Kunitz-type peptides [12,13]. These peptides have an ancient Kunitz fold formed by 56–58 a.a. and stabilized by three conservatively located disulfide bonds. Some of them are characterized by a unique and intriguing feature of dual functionality since they inhibit both proteases and ion channels [14,15,16,17,18]. Moreover, our recent results have demonstrated that sea anemone *Heteractis magnifica* (=*H. crispa*) Kunitz-type peptides have neuroprotective activity in different models of neurotoxicity in Neuro-2a neuroblastoma cells [19,20,21]. Pre-incubation of neuroblastoma cells with peptides followed by treatment with neurotoxins such as 6-OHDA, rotenone, paraquat as well as ATP resulted in an increase in cell viability via ROS production decrease (including NO). Nevertheless, the exact mechanism of cytoprotection has not been established. The P2X7 receptor was shown to be the intended target at least for two peptides, HCIQ4c7 and HMIQ3c1 [20].

In this work, we evaluated the ability of recombinant analogs of the sea anemone Kunitz-type peptides to influence ionotropic P2X7 purinergic receptor functioning in neurons and thereby protect cells from neurodegeneration. For this purpose, the MTT method to detect cell viability, spectrofluorimetry approach to detect Ca^2+^ influx and fluorescent dye YO-PRO-1 penetration count through P2X7 macropore were utilized. All recombinant peptides were tested, and for the most active peptides the binding to P2X7 by the SPR method was confirmed and interaction sites on the receptor were predicted by molecular docking.

## 2. Results

### 2.1. Expression and Purification of Recombinant Peptides

All studied peptides, APHC3, HCGS1.10, HCGS1.19, HCGS1.20, HCGS1.36, HCRG1, HCRG2, HCRG7, and HCRG21 were obtained as thioredoxin fusion proteins via expression in *Escherichia coli* using recombinant plasmids created on the base of the vector pET32b(+). The production of recombinant peptides was carried out in accordance with the previously used technique utilizing Ni^2+^-affinity chromatography followed by fusion protein cleavage by BrCN [18,21,22,23,24]. This approach was successfully applied for HCRG1, HCRG2, and HCRG7. The RP-HPLC retention times, molecular weight and ^1^H NMR spectra of APHC3, HCGS1.10, HCGS1.19, HCGS1.20, HCGS1.36, and HCRG21 peptides were identical to those described previously [18,21,22]. The final RP-HPLC results for the recombinant peptides HCRG1, HCRG2, and HCRG7 obtained for the first time in this work are shown in Figure S1. The yield of these peptides varied from 4.3 to 8.5 mg/L of the cell culture. The molecular weights of these peptides measured by the MALDI-TOF MS technique were 6195, 6148, and 6373 Da for HCRG1, HCRG2, and HCRG7, respectively, (Figure S1) and were in good accordance with the calculated value. The N-terminal amino acid sequence of HCRG1, HCRG2, and HCRG7 (15 a.a. residues) was determined by Edman automatic sequencing and appeared to be identical to that of HCRG1 and HCRG2 peptides [25], as well as an evaluation based on gene sequence for HCRG7. According to the ^1^H NMR spectra, the peptides have a pronounced spatial structure as evidenced by a wide chemical shift dispersion of amide hydrogens to the field of 8–10 ppm and the presence of resonance signals below 0 ppm (Figure S2).

### 2.2. Peptides Interaction with Neuronal Cells Neuro-2a

#### 2.2.1. Peptides Protect Cells from ATP-Induced Death

All recombinant peptides were tested in vitro for their ability to prevent the action of a high concentration of ATP, which triggers an inflammatory cascade accompanied by various processes in cells and leads to cell death. To exclude the self-toxicity of peptides on the cells investigated, their cytotoxicity was estimated using the MTT assay. All peptides did not show any toxic activity on Neuro-2a mouse neuroblastoma cells at a concentration of up to 100 μM.

**Figure 1:**
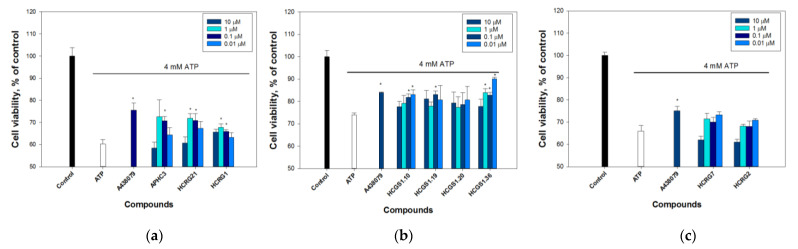
Cytoprotective activity of peptides on Neuro-2a cells treated with 4 mM ATP. Cellular viability was measured at 48 h after cells incubation with ATP alone/together with the peptides or A438079 as the standard P2X7 antagonist. Control cells were incubated in DMEM for 24 h, 37 °C, 5% CO_2_. The peptides in these experiments were divided into groups on microplates with their own controls, so they are separated in the graphs: (**a**) peptides APHC3, HCRG21, HCRG1; (**b**) peptides HCGS1.10, HCGS1.19, HCGS1.20, HCGS1.36; (**c**) peptides HCRG7, HCRG2. Data are presented as means ± SE (*n* = 3); * *p* < 0.05 compared with ATP alone.

To determine whether ATP-induced Neuro-2a cell death is due to P2X7 activation, the cells were pre-incubated with 10 μM A438079, a standard P2X7 blocker, and then with ATP for 48 h. The action of 4 mM ATP has been shown to cause significant cell death (Figure 1); the percentage of all dead cells was increased up to 40% compared to the number of cells incubated without ATP.

The viability of cells pre-incubated with 10 μM A438079 was increased by 18.2 ± 2.8% compared to cells incubated with ATP alone, indicating that this process was mediated by P2X7. Most tested peptides at concentrations less than 1 μM increased cell viability, but the most significant effect was measured for HCGS1.10 and HCGS1.36. Both peptides increased cell viability significantly at concentrations 0.01 μM up to the level reached by 10 μM A438079. However, the effect-concentration dependence for peptides was not clearly traced.

#### 2.2.2. Peptides Regulate ATP-Induced Calcium Influx

**Figure 2:**
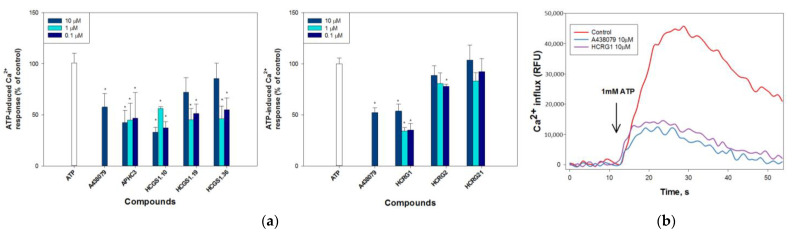
ATP-induced intracellular calcium increase in Neuro-2a cells. (**a**) Relative effect of compounds pre-incubation at concentrations 0.1–10 µM for peptides and 10 µM for P2X7 blocker A438079 on 1 mM ATP-induced Ca^2+^ level increase. (**b**) Representative trace of Fluo-8AM dye fluorescence intensity for untreated Neuro-2a cells (control) and pre-incubated 30 min cells with 10 µM HCRG1 or 10 µM A438079. The arrow shows the time of ATP administration. The peptides in these experiments were divided into groups on microplates with their own controls, so they are separated in the graphs. Data are presented as means ± SE (*n* = 3); * *p* < 0.05 to ATP alone data.

P2X7 receptors activated by ATP are known to act as nonselective cation channels; therefore, they are involved in the direct increase in the intracellular concentration of calcium ions ([Ca^2+^]i). To determine whether the peptides act directly on P2X7, we examined with the Fluo-8AM Ca^2+^ selective fluorescent probe, their ability to block Ca^2+^ influx when neuronal cells are exposed to high ATP concentrations. A strong sustained increase in [Ca^2+^]I was observed after the addition of 1 mM ATP (Figure 2) indicating purine receptor involvement in this process. The application of 10 µM A438079 led to a decrease in ATP-induced intracellular Ca^2+^ concentration by approximately 47% (Figure 2a). The addition of the peptides to the incubation medium did not lead to any changes in the intracellular Ca^2+^ content. Moreover, the simultaneous peptide addition together with ATP did not lead to a decrease in Ca-responses of cells to high concentrations of ATP, which may indicate the absence of a peptide additive effect.

To investigate the impact of P2Y receptors presented also in Neuro-2a cells, the cells were cultured in a Ca^2+^-free medium since metabotropic P2Y receptors lack the ability to provide calcium current through cellular membranes in contrast to P2Xs. Under these conditions, 1 mM ATP did not cause an increase in [Ca^2+^]I, thus P2Xs, not P2Ys, were responsible for a measured effect of ATP on Neuro-2a cells (Figure S3, Supplementary Information). The application of the Ca-selective ionophore ionomycin and the specific blocker of sarco/endoplasmic reticulum Ca^2+^-ATPase (SERCA), 2,5,-di(t-butyl) hydroquinone (BHQ), in a Ca-free medium led to a significant increase in [Ca^2+^]I (Figure S3, Supplementary Materials). Consequently, intracellular calcium depo is not involved in the Ca-response of cells to high concentrations of ATP.

The [Ca^2+^]I monitoring revealed that three peptides, APHC3, HCRG1, and HCGS1.10, at concentrations of 0.1–10 μM significantly reduced the calcium level by 53.2 ± 24.95% (0.1 μM APHC3), 57.5 ± 11.6% (0.1 μM HCRG1), and 43.8 ± 1.7% (0.1 μM HCGS1.10). Two peptides HCGS1.19 at concentrations of 0.1–1 μM and HCGS1.36 at a concentration of 0.1–1 μM were also able to reduce the calcium level by 48.7 ± 8.9/54.8 ± 10.8 and 45.12 ± 11.7/53.9 ± 12.5%, respectively; it was comparable to a 10 μM A438079 effect equal to 42–47% (Figure 2). The HCRG2 peptide insignificantly reduced the calcium level but at concentration of 0.1 μM only, while HCRG21 was not active at any of the tested concentrations.

#### 2.2.3. Peptides Reduce P2X7 Mediated Dye Uptake

**Figure 3:**
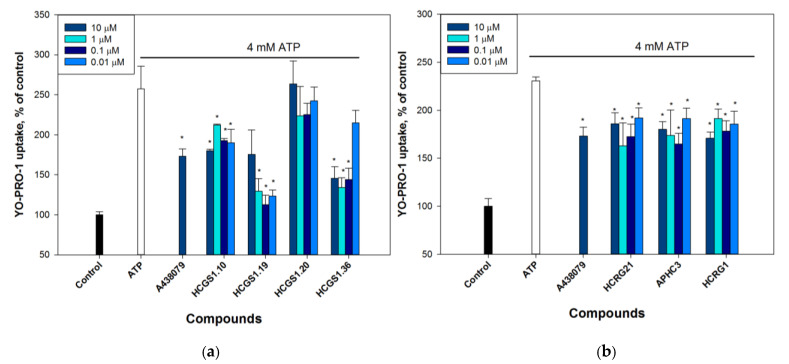
Uptake of fluorescent dye YO-PRO-1 by Neuro-2a cells after 4 mM ATP application. The 10 min pre-incubation with 0.01–10 μM peptides or 10 μM A438079 attenuated dye accumulation. The peptides in these experiments were divided into groups on microplates with their own controls, so they are separated in the graphs: (**a**) peptides HCGS1.10, HCGS1.19, HCGS1.20, HCGS1.36; (**b**) peptides HCRG21, APHC3, HCRG1. Data are presented as means ± SE (*n* = 3); * *p* < 0.05 compared with ATP alone.

Since the activation of P2X7 induces a large conductance pore formation followed by cell death, we used fluorescent dye YO-PRO-1 uptake by Neuro-2a cells to study the potential of peptides to influence P2X7 pore formation in cells. For this study, the active peptides mentioned above were selected. Pre-incubation of cells with 10 μM A438079 resulted in up to 32% inhibition of dye penetration following 4 mM ATP stimulus then compared to cells incubated with ATP alone. All studied peptides at concentrations ranging from 0.01 to 10 μM reduced ATP-induced uptake of YO-PRO-1 by Neuro-2a cells as well (Figure 3). Peptides HCGS1.36 and HCGS1.19 showed a stronger effect than A438079 reducing dye uptake to 47.8 ± 4.8 and 56.2 ± 4.5%, respectively. Thus, the studied peptides were shown to impact the ATP-induced negative effect on Neuro-2a cells through the P2X7 pore formation.

### 2.3. Interaction of Peptides with P2X7 Subunit

**Figure 4:**
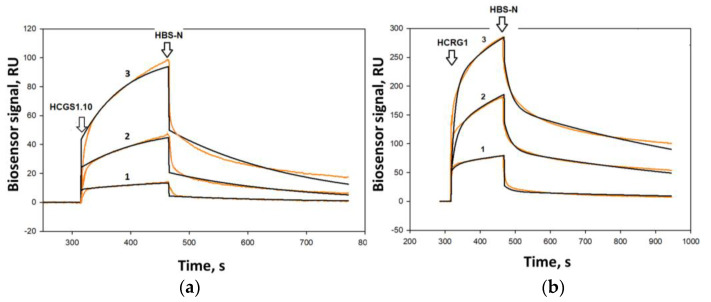
Sensorgrams of surface plasmon resonance binding obtained for immobilized P2X7 after HCGS1.10 (**a**) and HCRG1 (**b**) application at 25 °C by 10 µM concentration (curve 1), 25 µM (curve 2), 50 µM (curve 3). Fitting curves (theoretical model) drawn in black; Chi2 was calculated as 8.95 for HCGS1.10 and 6.75 for HCRG1. The time of peptides and HBS-N buffer injection is indicated by arrow.

To conclude that the studied peptides are able to interact with P2X7 directly, the surface plasmon resonance (SPR) method was used. For this purpose, the extracellular domain P2X7 (36.8 kDa) was immobilized on the optical biosensor chip of Biacore 3000 (12.3 ng/mm^2^). The dose-response curves were obtained for all studied peptides; however, the best affinity was found for HCGS1.10 and HCRG1 (Table 1) according to Biacore sensorgram data (Figure 4). The highest affinity was found for the HCRG1 peptide (*K_d_* 7.13 μM) which turned out to be more effective than previously studied peptides [20].

### 2.4. Influence of Peptides on P2X7 Expression in Neuro-2a Cells

**Figure 5:**
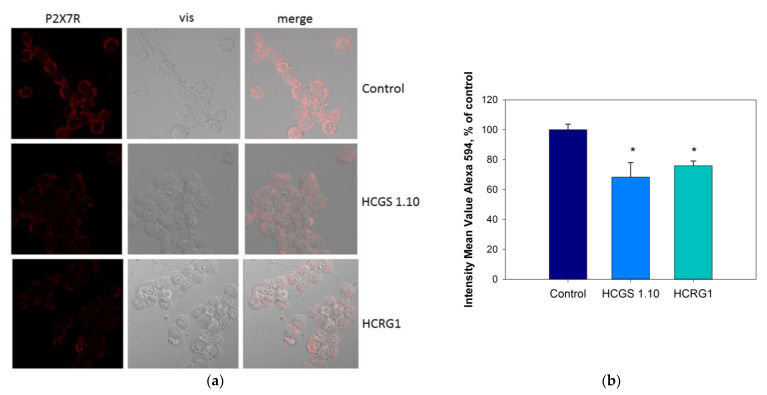
The P2X7 localization in Neuro-2a cells by antibodies to the P2X7. (**a**) Images obtained in confocal microscopy and (**b**) quantification result of fluorescence intensity after pre-incubation of cells with peptides for 24 h with peptides HCGS1.10 and HCRG1 at 10 µM concentration. Data are presented as means ± SE (*n* = 3); * *p* < 0.05.

Immunofluorescence studies of the P2X7 receptor and its possible internalization in the neuronal cells were studied by P2X7-specific antibodies. Analysis of confocal images of the cells showed the receptor presence on the cell membrane. The cell incubation with peptides at a concentration of 10 μM for 24 h resulted in a significant decrease in fluorescence. Analysis of the fluorescent image of the cells showed that the decrease was 31.6 ± 9.7 and 24.1 ± 3.2% for HCGS1.10 and HCRG1, respectively (Figure 5).

### 2.5. Molecular Docking

**Figure 6:**
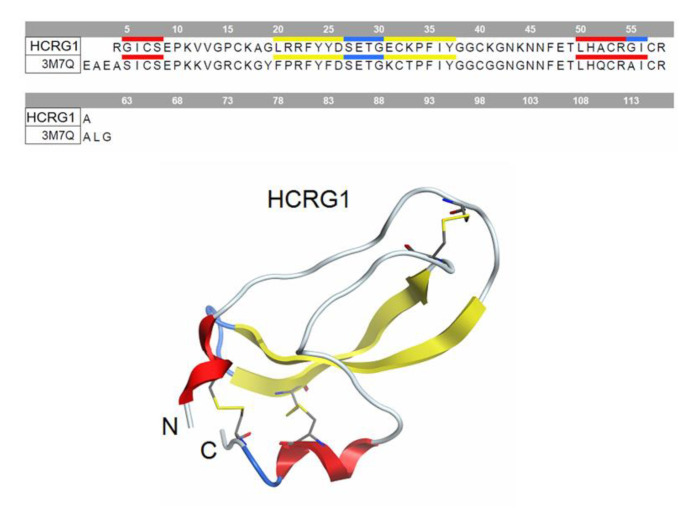
The homology model of the peptide HCRG1 represented as ribbon diagram and the alignment of the amino acid sequence of HCRG1 and Kunitz type serine protease inhibitor-1 (3M7Q).

The putative HCRG1 peptide’s binding sites on mP2X7 homotrimeric extracellular domain were predicted by the methods of structural bioinformatic and molecular docking. The HCRG1 peptide was chosen as a ligand that presented the highest affinity in the SPR. The HCRG1 homology model was generated on the base of the crystal structure of the Kunitz-type serine protease inhibitor-1 (SHPI-1) from the sea anemone *Stichodactyla helianthus* (the amino acid sequence identity of the model and template was 73% to PDB ID 3M7Q_B) [26] is presented on Figure 6.

**Figure 7:**
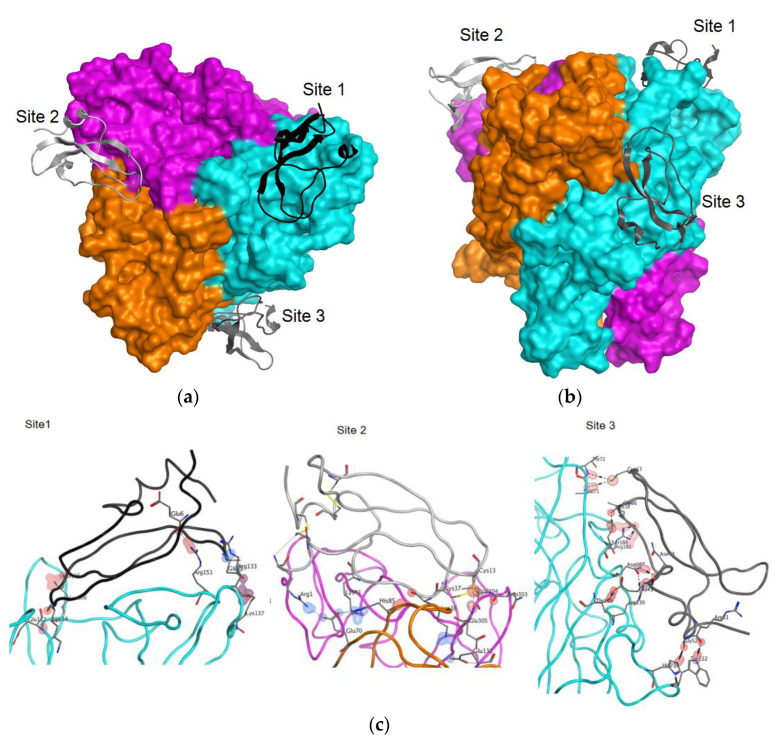
Molecular docking of the peptide HCRG1 and the extracellular domain of the mP2X7 homotrimer. The molecular surface of the mP2X7 extracellular domain is shown in orange, pink and turquoise for the corresponding monomer. (**a**) Top view of the extracellular domain along the central axis. (**b**) Side view of the extracellular domain. (**c**) The HCRG1 peptide contacts with P2X7 at binding sites 1, 2 and 3. The structure of P2X7 fragments is shown in turquoise, pink, and orange. The amino acid residues forming the contacts are shown as sticks. The atoms involved in the actual contact are highlighted by shells rendered around the interacting atoms. The peptide HCRG1 structure at sites 1–3 is shown in black, light gray, and dark gray, respectively.

The 3D-structure of the mP2X7 homotrimer was simulated, and the extracellular domain of the trimer was used for docking with HCRG1. The structures of 100 complexes of the peptide with the extracellular domain of the mP2X7 homotrimer were calculated. According to the data obtained, three binding sites with the best docking scoring were localized on the top of the extracellular domain and on the side of the monomer were found (Figure 7, Table 2).

The peptide/mP2X7 interaction can be realized predominantly by two extended loops, which in the case of Kunitz peptides form the main binding site (a.a. in positions 9–17) and the weak interactions site (a.a. in positions 34–39) to proteases [20,26]. Such residues as Cys13, Lys14, and Gly16 of the main binding site as well as Gly36 and Lys38 of the weak interactions site are involved in the complex formation. In site 1, the strongest interaction was found between Lys137 and Glu25, and between Lys306 and Gly16. In site 2, the strongest interaction was between Glu70 and Lys41. In site 3, the strongest interaction was between Arg230 and Asn43.

**Figure 8:**
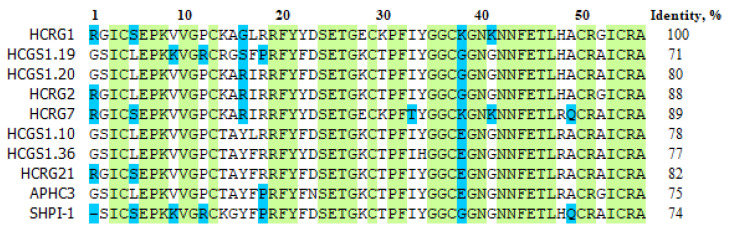
Multiple alignment of sea anemones Kunitz-type peptides using in the research: HCRG1 (UniProtKB ID: C0HJU6), HCRG2 (C0HJU7), HCRG7, HCRG21 (P0DL86), HCGS1.10 (P0DV03), HCGS1.19 (P0DV04), HCGS1.20 (P0DV05), HCGS1.36 (P0DV06), and APHC3 (C0HJF3) from *H. magnifica* (=*H. crispa*) and SHPI-1 (P31713) from *Stichodactyla helianthus*.

The peptides studied in this work share a sufficiently high percentage of sequence identity up to 89%, including amino acid residues involved in the formation of complexes at sites 1–3 (Figure 8). Unlike other peptides, HCRG1 has Gly at position 16, whose contribution to the complex formation in site 1 is significant (Table 2).

## 3. Discussion

The role of ATP as an extracellular messenger in the processes of neurodegeneration cannot be overestimated. During brain injury, ischemia, inflammation and cell death, a significant amount of intracellular ATP is released and, as a result, the purinergic signaling pathway is stimulated, in which P2X7 receptors play a significant role. P2X7 receptors are ligand-gated ion channels that are activated by ATP and are present in a variety of cells, including cells of the immune system, neurons, and microglia in the central nervous system [27]. Unlike other purinergic receptors of the P2X family, this type of receptor is activated by high concentrations of ATP (1 mM and more) and is characterized by a long desensitization time [28]. P2X7 has now been proven to play an important role and represent an important therapeutic target in many pathological conditions. P2X7 is known to be overexpressed and overactivated by pathologies of the central nervous system, such as Alzheimer’s disease, Parkinson’s disease, and Huntington’s disease [7]. The therapeutic potential of P2X7 agonists and antagonists is currently being intensively studied for the treatment of a number of diseases, including chronic neuropathic and inflammatory pain, cancer and intracellular infections as well as neuroinflammation and neurodegenerative diseases.

The Neuro-2a cell model that has been extensively characterized in the literature is a mouse neuroblastoma expressing native P2X7 [29,30]. In our study, we confirmed by immunocytochemistry and subsequent confocal microscopy that P2X7 receptors are localized on the surface of this cell type. We found that high concentrations of ATP cause a sharp increase in the concentration of Ca^2+^ in the cell cytoplasm. The dynamics of [Ca^2+^]_i_ changes under the ATP action on cells corresponds to that observed in cells upon P2X7 activation. The absence of a Ca^2+^ response to ATP in a calcium-free medium, as well as an increase in the release of Ca^2+^ from intracellular depo upon subsequent application of the Ca-selective ionophore ionomycin and/or the specific SERCA blocker, BHQ, means that high concentrations of ATP activate P2X7 in the biomembranes of Neuro-2a cells rather than intracellular receptors. Additional evidence for the P2X7 activation under the action of high ATP concentrations is an increase in the entry of the low molecular weight marker YO-PRO-1 into the cytoplasm indicating the formation of macropores in cell membranes as well as the sensitivity of calcium and YO-PRO-1 transport to the competitive P2X7 antagonist A438079. Incomplete blocking of the receptor by A438079 is common. For example, in pancreatic ductal adenocarcinoma cells, this antagonist had very modest effects on ATP-induced pore formation and cell death [31].

In our research, nine recombinant peptides, analogs of sea anemone Kunitz-type peptides, were tested for their ability to reduce ATP-induced cell death through interaction with P2X7. The ability of the peptides to influence the cytotoxic effect of ATP was evaluated on Neuro-2a mouse neuroblastoma cells the death of which was induced by high concentrations of ATP. It is known that ATP-induced and P2X7-mediated formation of macropores leads to the formation of a large amount of ROS which triggers apoptosis and subsequent cell death [32,33,34]. We have shown that the peptides HCRG1, HCGS1.10, HCGS1.19, HCGS1.36, APHC3 and HCRG21 partially protected neuroblastoma cells from ATP-induced death during long-time application of adenosine triphosphate.

As a short-term effect for HCRG1, HCGS1.10, HCGS1.19, HCGS1.36, APHC3 and HCRG21, a significant effect on Ca^2+^ influx into cells regulating the entry of low molecular weight fluorescent dye YO-PRO-1 into cells was observed. Moreover, this effect is not associated with the additive effect and interaction of peptides with ATP in the extracellular environment. Thus, the regulation of the ATP-induced entry of calcium ions and YO-PRO-1 into cells together with the cytoprotective effect of peptides indicates an influence on the functioning of P2X7, the activation of which leads to cell death. In addition, analysis of the fluorescent image of cells specifically stained with antibodies against P2X7 showed that HCRG1 and HCGS1.10 significantly reduced the amount of P2X7 in Neuro-2a cells. This may indicate a partial suppression of this receptor expression in neuroblastoma under the peptide influence which may also contribute to the manifestation of the cytoprotective properties of peptides.

All peptides that significantly protected Neuro-2a cells from the ATP cytotoxic action showed a “bell-shaped” or inverse “U-shaped” dose dependence rather than a linear dose dependence [35,36,37]. The peptide highest concentration tested (10 μM) was largely ineffective despite significant efficacy at lower concentrations. The most likely mechanism for this phenomenon in the Neuro-2a cell model is ligand-induced down-regulation of the receptor at high ligand concentrations.

Based on the obtained data we selected the two most active peptides, HCRG1 and HCGS1.10, for which their selective binding to P2X7 subunits and the formation of stable complexes were demonstrated by SPR. This interaction is a direct confirmation of the ability of the active peptides to specifically interact with P2X7 subtype purine receptors which can lead to an effect on their functioning. The parameters of peptide binding to P2X7 immobilized on a chip calculated using the SPR method may differ from those observed in experiments with live cultured cells since the conditions for these two types of studies are significantly different.

Using the molecular docking approach, we calculated 100 structures of complexes of the most active HCRG1 peptide with the extracellular domain of the mP2X7 homotrimer. Three of them with HCRG1 localized at the top of the extracellular domain and on the monomer side were chosen as structures with the best docking scoring. We suggested that the regulatory effect of the peptides on ATP-induced activation of P2X7 may be associated with the prevention of the conformational rearrangement of the channel from a closed to an open state. Site 1 is more preferred, and the best activity of HCRG1 compared to other peptides may be due to the presence of Gly at position 16, unlike other peptides.

The studied peptides are analogs of sea anemone Kunitz-type peptides and attract our attention due to their wide spectrum of biological activity and ability to interact with important molecular targets. It has been shown that the APHC3 peptide having cytoprotective properties is an effective modulator of the TRPV1 receptor that differently affects the channel depending on the nature and strength of activation stimuli with EC_50_ ~18 nM. This peptide revealed potent activity in ex vivo and in vivo models of inflammatory diseases. Peptide HCRG21 was reported as a complete blocker of capsaicin-induced currents of TRPV1 with IC_50_ ~ 6.9 μM. Both peptides had significant anti-inflammatory and analgesic activity [14,17,18,21,38,39,40]. It is assumed that HCGS1.19, HCGS1.20, and HCGS1.36 peptides significantly inhibit histamine H1-receptors in macrophage cells [23,24]. In addition, HCRG1 and HCRG2 peptides were found to be nonselective potassium channel blockers with an IC_50_ of 12–400 nM for Kv1.1, Kv1.2, Kv1.3 and Kv1.6 isoforms as well as insect Shaker IR [16,25].

To date, over 50 different proteins have been identified that physically interact with P2X7. However, few of these interactions have been confirmed in independent studies and for most of these proteins, the interaction domains and physiological consequences of the interactions are poorly described [41,42]. In the present study we have shown for the first time that a number of recombinant peptides, analogs of Kunitz-type peptides from sea anemones, are able to influence, among other things, purinergic P2X7 receptors in neuronal cells. This indicates their pharmacological potential as neuroprotective compounds. In summary, we can conclude that the studied peptides can become the basis for creating new molecular tools in the study of the P2X7 receptor function and for the production of new drugs with analgesic and neuroprotective effects.

## 4. Materials and Methods

### 4.1. Expression and Isolation of Kunitz-Type Recombinant Peptides

The pET32b(+)/*hcrg1*, pET32b(+)/*hcrg2* constructions were synthesized by JSC Eurogen (Moscow, Russia) while pET32b(+)/*hcrg7* and were made by cloning of gene *hcrg7* from cDNA library to pET32b(+) at the EcoR1 and XhoI restriction sites. The Kunitz-type peptides, APHC3, HCGS1.10, HCGS1.19, HCGS1.20, HCGS1.36, and HCRG21, as well as new HCRG1, HCRG2, and HCRG7, were obtained as described in [18,21,22,23,24].

### 4.2. Mass Spectrometry Analysis

MALDI-TOF MS spectra of peptides were recorded using an Ultraflex III MALDI-TOF/TOF mass spectrometer (Bruker, Bremen, Germany) with a nitrogen laser (Smart Beam, 355 nm), reflector and potential LIFT tandem modes of operation. Sinapinic acid was used as a matrix. External calibration was employed using a peptide InhVJ with *m*/*z* 6107 [43] and its doubly-charged variant at *m*/*z* 3053.

### 4.3. Amino Acid Sequence Determination

Recombinant peptides HCRG1, HCRG2, and HCRG7 were reduced and alkylated with 4-vinylpyridine as described in [25]. Alkylated peptides were desalted by RP-HPLC using a Nucleosil C_18_ column (4.6 × 250 mm) (Sigma Aldrich, St. Louis, MO, USA). The solvents A and B were 0.1% TFA in water and in acetonitrile, respectively. The chromatographic runs were performed using a 10–70% gradient of solvent B over 160 min at a flow rate of 0.5 mL/min. UV detection was monitored at 214 nm.

The N-terminal amino acid sequences were determined on an automated sequencer protein Procise 492 Clc (Applied Biosystems, Waltham, MA, USA).

### 4.4. One-Dimensional NMR Spectroscopy

The ^1^H NMR spectra of peptides were acquired at 30 °C on a Bruker Avance III 700 MHz spectrometer (Bruker Biospin, Billerica, MA, USA) equipped with a triple resonance z-gradient TXO probe. Peptides were dissolved in 90% H_2_O/10% D_2_O (Deutero GmbH, Kastellaun, Germany) at a concentration of 1.5–2 mg/mL. Excitation sculpting with gradients [44] was applied to suppress strong solvent resonance, and the chemical shift of their signal was arbitrarily chosen as 4.7 ppm. TopSpin 3.6 (Bruker Biospin, Billerica, MA, USA) was used for spectrum acquisition and processing.

### 4.5. Cell Culture

Neuro-2a neuroblastoma cells (ATCC^®^ CCL-131™) were obtained from American Type Culture Collection (Manassas, VA, USA). The cells were cultivated in DMEM medium (BioloT, St. Petersburg, Russia) supplemented with 10% of fetal bovine serum (BioloT, St. Petersburg, Russia) and 1% penicillin/streptomycin (BioloT, St. Petersburg, Russia) at 37 °C and 5% CO_2_. Initially, cells were incubated in cultural flasks until sub-confluent (~80%). For testing, Neuro-2a cells were seeded in 96-well plates and experiments were started after 24 h.

### 4.6. Analysis of Cytotoxic Activity

Peptides at different concentrations were added in Neuro-2a cell (1 × 10^4^ cells per well of 96-well plate). Then cells were incubated for 24 h at 37 °C and 5% CO_2_. Then 10 µL of stock solution of MTT (3-(4,5-dimethylthiazol-2-yl)-2,5-diphenyltetrazolium bromide) (Sigma-Aldrich, St. Louis, MO, USA) (5 mg/mL) was added to each well, and the microplate was incubated for 4 h. Thereafter, 100 µL of SDS-HCl solution was added to each well, followed by 18 h incubation. The absorbance of the converted formazan dye was measured using a Multiskan FC microplate photometer (Thermo Scientific, Waltham, MA, USA) at a wavelength of 570 nm. All experiments were repeated in triplicate.

### 4.7. Cytoprotection Activity Assay

The cytoprotective effect of peptides was assessed in Neuro-2a cells using the MTT assay [45]. In brief, an aliquot (180μL) of cell suspension with a nominal cell density of 3 × 10^4^ cells/well was dispensed into 96-well plates. The cells were incubated for 24 h in a humidified atmosphere containing 5% CO_2_ to allow cell attachment. At the end of the preincubation period, ATP (Sigma-Aldrich, St. Louis, MO, USA) was added to the cells at a concentration of 4 mM. Studied peptides were added to the cells at concentrations of 0.1, 1.0 or 10.0 μM for 1h prior to the addition of ATP. After incubation for 48 h at 37 °C cell viability was detected using the MTT as described previously. The standard P2X7 inhibitor, A438079 (Sigma-Aldrich, St. Louis, MO, USA), at a concentration of 10 μM was used as a positive control.

### 4.8. Ca^2+^Influx Measurement

Neuro-2a cells were seeded in 96-well plates at a density of 4 × 10^4^ cells/well and incubated in DMEM for 24 h at 37 °C, 5% CO_2_. Then the cells were washed twice with culture medium and loaded with 5 μM Fluo-8 AM (Abcam, Cambridge, UK) dye and 1 μM Pluronic^®^ F-127 (Sigma-Aldrich, St. Louis, MO, USA), and incubated for 40 min at 37 °C in HBSS: 140 mM NaCl, 5 mM KCl, 0.8 mM MgCl_2_, 2 mM CaCl_2_, 10 mM glucose, 10 mM HEPES, pH 7.4. Then the cells were washed two times with the same solution but without the fluorescent dye and treated with the studied peptides for 30 min at RT in the dark. In some experiments Ca^2+^-free medium was used: 140 mM NaCl, 5 mM KCl, 0.8 mM MgCl_2_, 10 mM glucose, 10 mM HEPES, 5 mM EGTA, pH 7.4. ATP (1 mM final concentration) was added using a microinjector (20 μL/well) 10 s later the baseline recording and the readings were taken up to 50 s in 1 s intervals. The standard P2X7 inhibitor, A438079 (Sigma-Aldrich, St. Louis, MO, USA), was used as inhibitory control. Ionomycin (Sigma-Aldrich, St. Louis, MO, USA) and BHQ were used to generate a generic calcium signal in cells. 2,5,-di(t-butyl) hydroquinone (BHQ) was synthesized and kindly provided by Dr. Polonik S.N. (PIBOC FEBRAS). Fluo-8 was excited at 485 nm, and the emission at 520 nm was measured with a PHERAstar^®^FS plate reader (BMG LABTECH, Ortenberg, Germany). The data were processed by MARS Data Analysis v. 3.01R2 (BMG Labtech, Ortenberg, Germany). Mean values and standard error of peak height calcium were calculated.

### 4.9. YO-PRO-1 Uptake Measurement

Neuro-2a cells were seeded in 96 well plates at a density of 4 × 10^4^ cells/well and incubated in DMEM for 24 h, 37 °C, 5% CO_2_. Then the cells were washed once with HBSS and filled with 180 μL of the same buffer. YO-PRO-1 (Sigma-Aldrich, St. Louis, MO, USA, 5 μM final concentration) was loaded into the wells, the cells were incubated for 15 min at 37 °C. Further, the studied peptides were added to the cells at concentrations of 10, 1, 0.1 and 0.01 μM and incubated for 10 min at 37 °C. After that ATP (4 mM final concentration) was added and the plates were incubated for an additional 10 min. The standard P2X7R inhibitor, A438079 (Sigma-Aldrich, St. Louis, MO, USA), was used as inhibitory control. Then the cells were washed three times with the buffer solution and fluorescence intensity was measured with PHERAstar FS plate reader (BMG, LABTECH, Ortenberg, Germany) at λex = 485 nm and λem= 520 nm in the “End Point” mode. The effectiveness of compounds was evaluated relative to control without ATP.

### 4.10. Surface Plasmon Resonance

SPR analysis was performed at 25 °C using a Biacore^®^ 3000 optical biosensor (GE Healthcare, Chicago, IL, USA) and CM5 sensor chips. HBS-N (10 mM HEPES, 150 mM NaCl, pH 7.4) (Cytiva, Chicago, IL, USA) was used as a working buffer for the immobilization of hP2X7 (47–334 a.a., extracellular domain, 36.8 kDa) (abx167020 Abbexa LTD, Cambridge, UK). The carboxyl groups of the dextran biosensor chip were activated for 5 min by introducing a 1:1 mixture of 0.2 M 1-ethyl-3-(3-dimethylaminopropyl)carbodiimide hydrochloride (EDC) and 0.05 M N-hydroxysuccinimide (NHS) at a rate of 5 µL/min followed by a 1 min wash with HBS-N buffer at the same flow rate. Next, hP2X7 (15 μg/mL) in 10 mM sodium acetate (pH 5.0) was injected into the working channel of the biosensor for 10 min at a flow rate of 5 μL/min. The final level of immobilization was 12300 RU (12.3 ng of protein). The control channel without immobilized P2X7R was used to correct the effects of the nonspecific binding of analytes to the chip surface.

Lyophilized peptides were prepared as 100 μM stock solutions in HBS-N buffer. Experimental peptide samples were prepared in the range of 5–100 μM. The samples were injected through the channels of the biosensor (working and reference) at a flow rate of 10 μL/min for 6 min. The dissociation of the formed peptide/P2X7 complexes was recorded for at least 6 min from the moment of injection at the same flow rate after the injection of the sample. After each cycle of the biosensor, the bound analyte was removed by double injection of the regenerating solution (2 M NaCl, 1% CHAPS) at a flow rate of 30 μL/min for 30 s.

SPR sensorgrams were processed in Biacore 3000 Evaluation Software v.1.0 (GE Healthcare, Chicago, IL, USA) and BIAevaluation Software v. 4.1.1 (GE Healthcare, Chicago, IL, USA) using “1:1 binding (Langmuir)” and “two-state binding (conformational change)” data processing models.

The 1:1 (Langmuir) binding model is a 1:1 interaction model between a compound (C) and an immobilized protein (P) and is equivalent to the Langmuir isotherm for surface adsorption: C + P ↔ CP.

The two-state binding (conformational change) model describes the 1:1 binding of a compound (C) to an immobilized protein (P) followed by a conformational change in the complex (CP ↔ CP*). It is assumed that the conformationally changed complex can dissociate only by the reverse conformational change: C + P ↔ CP ↔ CP*. The final kinetic parameters were obtained from the models that best agree with the experimental curves for the minimum of the obtained value of chi2. The equations describing the models used are as follows:

(1) 1:1 (Langmuir) binding:

*K_d_* = k_off_/k_on_,

where *K_d_* is the equilibrium dissociation constant, k_off_ is the dissociation rate constant, k_on_ is the association rate constant.

(2) Reaction of two states (with conformational change):

*K_d_* = k_off1_/k_on1_ × (1 + k_on2_/k_off2_) − 1,

where *K_d_* is the equilibrium dissociation constant, k_off1_ is the dissociation rate constant, k_on1_ is the association rate constant, k_on2_ is the direct association rate constant for the CP ↔ transition CP*, k_off2_ is the inverse dissociation rate constant for the CP ↔ CP* transition.

The model describing the interaction was chosen according to the lowest chi2 parameter’s value. Chi2 is a measure of the average squared residual between the experimental data and the fitted curve.

### 4.11. Molecular Modeling

The 3D-structures of the HCRG1 peptide were generated on the base of the crystal structure of a Kunitz type serine protease inhibitor-1 from the Caribbean sea anemone *Stichodactyla helianthus* (PDB ID 3M7Q_B) [26] with the Protein Homology module of the Molecular Operating Environment (MOE; 2020.09; Chemical Computing Group ULC, 1010 Sherbrooke St. West, Suite #910, Montreal, QC, Canada, H3A 2R7, 2020.) program using Amber10:EHT potential and 3D-protonation for structure optimization. Homology models of the mouse P2X7 monomer (UniProt ID Q9Z1M0) and trimer in the closed form were built with the Homology module of the MOE program using as a template, the closed-form structure of the rat P2X7 (PDB ID 6U9V) as described before [46]. Energy minimization of the mP2X7 model with the force field Amber10:EHT and 3D-protonation were carried out using the MOE program. The extracellular domain of the trimer was used for docking with the peptide. Molecular docking of the peptide to the model of mP2X7R trimer extracellular domain was carried out using the Protein-Protein Dock module of the MOE program. The structures of 100 complexes of the peptide with the extracellular domain were calculated. Analysis of peptide contacts with the extracellular domain of mP2X7 was carried out using the Protein contacts module of the MOE program for the three complexes with the lowest energy.

### 4.12. Immunocytochemistry

Neuro-2a cells on coverslips were washed once with PBS, fixed with cold 100% methanol for 5 min in an ice bath, and then washed (3 × 5 min) with PBS. For P2X7 receptor immunocytochemistry, cells were blocked with 10% bovine serum in PBS containing 0.1% Triton X-100 (1 h) and incubated with specific to P2X7 primary antibodies (1:1000; orb35965 Biorbyt Ltd., Cambridge, UK) overnight at 4 °C. Cells were washed with PBS (3 × 10 min) and incubated with Alexa Fluor 594-conjugated goat anti-rabbit secondary antibodies (1:200, Abcam plc, Cambridge, UK) for 2 h at room temperature. Cells were washed in PBS (3 × 10 min) before mounting. In control experiments, the primary antibody was omitted, and immunostaining was never observed. Neuro-2a cell fluorescence was studied using an LSM 710 META confocal laser scanning microscope (Carl Zeiss, Göttingen, Germany) equipped with an Ar laser with an effective power of 30 mW. The obtained images were analyzed using the ZEN 2.3 (blue edition) software (Carl Zeiss, Göttingen, Germany).

### 4.13. Statistics

All data were obtained in three independent replicates and calculated values were expressed as mean ± standard error of the mean (SEM). The Student’s *t*-test was performed using SigmaPlot 14.0 (Systat Software Inc., San Jose, CA, USA) to determine statistical significance.

## Figures and Tables

**Table 1:** Kinetic and equilibrium parameters of the P2X7/peptide complex.

Peptides	k_on1_, mole^−1^c^−1^	k_on2_, mole^−1^c^−1^	k_off1_, c^−1^	k_off2_, c^−1^	Kd, M	Evaluation Model
HCRG1	(9.20 ± 1.10) × 10^2^	(8.5 ± 1.0) × 10^−3^	(4.37 ± 0.57) × 10^−2^	(1.5 ± 0.20) × 10^−3^	7.13 × 10^−6^	Binding with two states
HCGS1.10	(1.71 ± 0.19) × 10^2^		(4.5 ± 0.52) × 10^−3^		2.63 × 10^−5^	Binding 1:1 (Langmuir 1:1)

The table shows the average values of the parameters ± standard deviation, *n* = 3.

**Table 2:** Amino acid contacts of HCRG1 with the mP2X7 homotrimer extracellular domain.

Site	Type *	ChainA	PosA	SetA	ChainB	PosB	SetB	Energy	Dist
	h	mm-trim-.A	65	Glu112	HCRG1	14	Lys14	−0.700	3.354
	i	mm-trim-.A	86	Arg133	HCRG1	25	Glu25	−1.505	3.609
Site 1	ih	mm-trim-.A	90	Lys137	HCRG1	25	Glu25	−8.017	2.393
	h	mm-trim-.A	104	Arg151	HCRG1	6	Glu6	−3.600	2.821
	h	mm-trim-.A	259	Lys306	HCRG1	16	Gly16	−7.800	2.976
	i	mm-trim-.B	23	Glu70	HCRG1	1	Arg1	−4.886	2.940
	i	mm-trim-.B	23	Glu70	HCRG1	41	Lys41	−5.965	2.800
	h	mm-trim-.B	38	His85	HCRG1	38	Lys38	−1.700	3.268
Site 2	i	mm-trim-.B	65	Glu112	HCRG1	38	Lys38	−2.364	3.385
	h	mm-trim-.C	256	Asn303	HCRG1	13	Cys13	0.300	3.246
	h	mm-trim-.C	257	Val304	HCRG1	13	Cys13	−0.800	2.918
	h	mm-trim-.C	258	Glu305	HCRG1	13	Cys13	−2.600	3.125
	h	mm-trim-.A	24	Val71	HCRG1	13	Cys13	−0.800	4.188
	h	mm-trim-.A	136	Arg183	HCRG1	38	Lys38	−3.600	2.968
	h	mm-trim-.A	137	Ser184	HCRG1	36	Gly36	−1.400	2.696
Site 3	h	mm-trim-.A	140	Asn187	HCRG1	43	Asn43	−1.900	2.833
	h	mm-trim-.A	142	Thr189	HCRG1	45	Glu45	−1.700	2.615
	h	mm-trim-.A	172	His219	HCRG1	52	Gly52	−3.800	2.783
	h	mm-trim-.A	175	Trp222	HCRG1	51	Arg51	−3.500	2.880
	h	mm-trim-.A	183	Arg230	HCRG1	43	Asn43	−6.900	2.809

* Type of contact: h—hydrogen bond contact; i—ionic bond contact; ih—ionic-hydrogen bond contact.

## Data Availability

Not applicable.

## Supplementary Materials

The following supporting information can be downloaded at: https://www.mdpi.com/article/10.3390/md21030192/s1, Figure S1: The RP-HPLC elution profiles of Kunitz peptides: HCRG1 (a), HCRG2 (b), and HCRG7 (c), on a Jupiter C4 column (Phenomenex, USA), equilibrated by 0.1% TFA, in a gradient of acetonitrile concentration (0–70%) for 70 min at 1.5 mL/min. MALDI mass spectra of the average molecular weight of peptides are shown in the insert. Black solid lines accentuate fractions from which the peptides were isolated. Figure S2: 1H NMR spectra of HCRG1 (a) and HCRG7 (b). Figure S3. Representative traces of [Ca^2+^]i increase induced by ionomycin (10 μM) in the presence of ATP 1 mM in Ca-free medium in Neuro-2a cells (a). Influence of SERCA blocker BHQ (50 μM) in Ca-free medium on Ca^2+^ influx caused by ATP (1 mM) in Neuro-2a cells (b). The data are presented as the m ± SE values (*n* = 6).
