# Supplementary material for: Recombinant Analogs of Sea Anemone Kunitz-Type Peptides Influence P2X7 Receptor Activity in Neuro-2a Cells
Source: Mar Drugs. 2023 Mar 20;21(3):192. doi: 10.3390/md21030192 (PMC10053369; doi:10.3390/md21030192)
Supplement: Supplementary file 1 [file marinedrugs-21-00192-s001.zip › marinedrugs-2299532-supplementary.pdf]

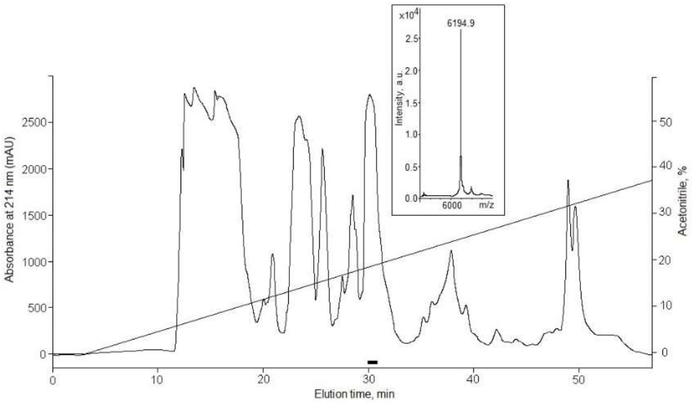

(**a**)

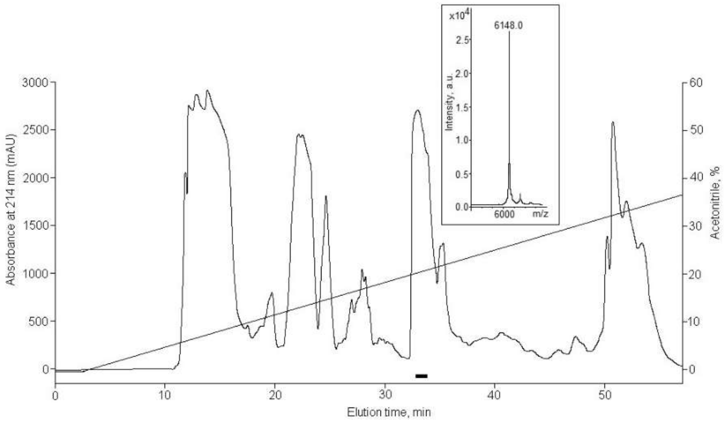

(**b**)

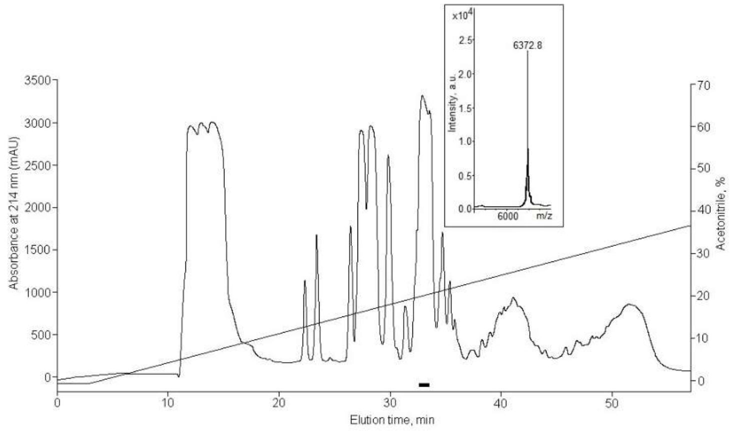

(**c**)

Figure S1. The RP-HPLC elution profiles of Kunitz peptides: HCRG1 (**a**), HCRG2 (**b**), and HCRG7 (**c**), on a Jupiter C4 column (Phenomenex, USA), equilibrated by 0.1% TFA, pH 2.2, in a gradient of acetonitrile concentration (0–70%) for 70 min at 1.5 mL/min. MALDI mass spectra of average molecular weight of peptides are shown in insert. Black solid lines accentuate fractions from which the peptide was isolated.

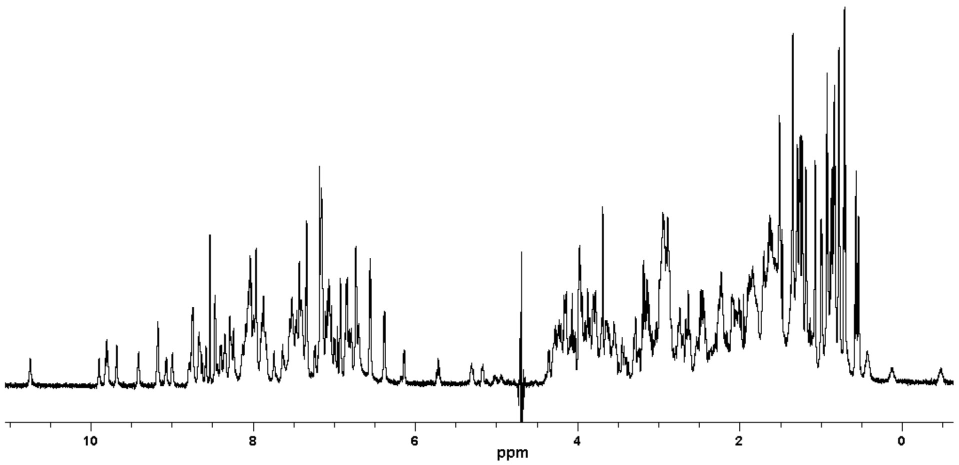

(**a**)

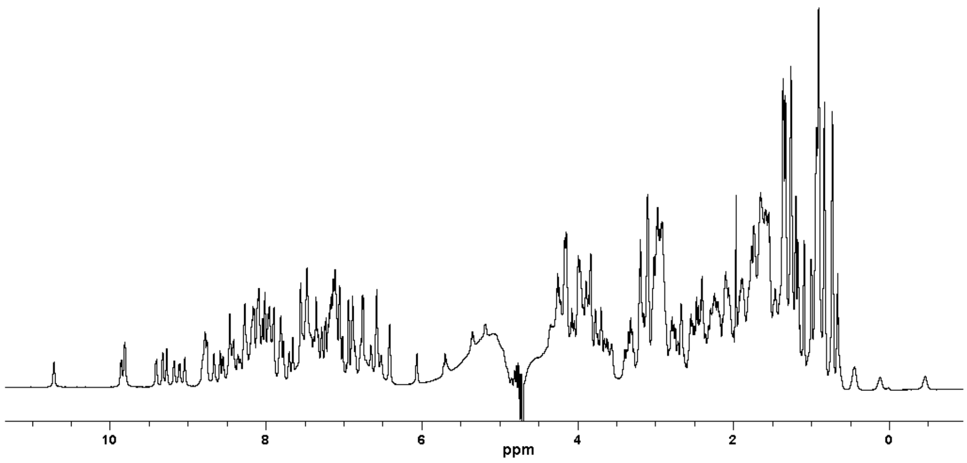

(**b**)

Figure S2. 1H NMR spectra of HCRG1 (**a**) and HCRG7 (**b**).

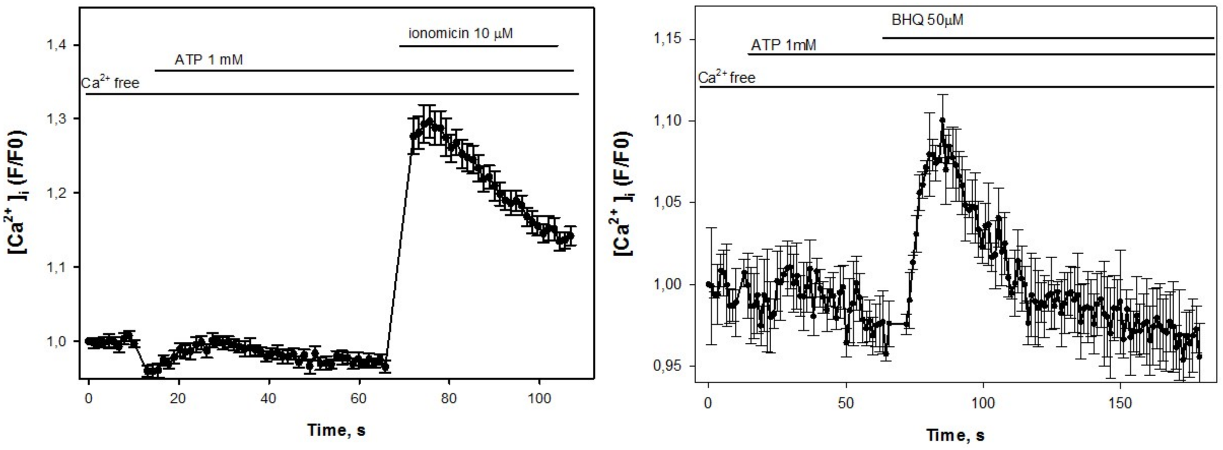

(a) (b)

Figure S3. Representative traces of $[Ca^{2+}]_i$ increase induced by ionomycin (10 μM) in the presence of ATP 1 mM in Ca-free medium in Neuro-2a cells (a). Influence of SERCA blocker BHQ (50 μM) in Ca-free medium on Ca^{2+} influx caused by ATP (1 mM) in Neuro-2a cells (b). The data are presented as the m ± SE values (n = 6).
